# The role of beta cell heterogeneity in islet function and insulin release

**DOI:** 10.1530/JME-18-0011

**Published:** 2018-04-16

**Authors:** Daniela Nasteska, David J Hodson

**Affiliations:** 1 Institute of Metabolism and Systems Research (IMSR)University of Birmingham, Edgbaston, UK; 2 Centre for EndocrinologyDiabetes and Metabolism, Birmingham Health Partners, Birmingham, UK; 3 COMPARE University of Birmingham and University of Nottingham MidlandsBirmingham, UK

**Keywords:** insulin secretion, diabetes II, metabolism, pancreatic beta cell

## Abstract

It is becoming increasingly apparent that not all insulin-secreting beta cells are equal. Subtle differences exist at the transcriptomic and protein expression levels, with repercussions for beta cell survival/proliferation, calcium signalling and insulin release. Notably, beta cell heterogeneity displays plasticity during development, metabolic stress and type 2 diabetes mellitus (T2DM). Thus, heterogeneity or lack thereof may be an important contributor to beta cell failure during T2DM in both rodents and humans. The present review will discuss the molecular and cellular features of beta cell heterogeneity at both the single-cell and islet level, explore how this influences islet function and insulin release and look into the alterations that may occur during obesity and T2DM.

## Introduction

Type 2 diabetes mellitus (T2DM) is a complex metabolic disorder which occurs when genetically susceptible individuals are exposed to a permissive environment. It currently affects ~10% of the adult population (Ezzati & World Health O 2004) and represents an escalating healthcare crisis. T2DM not only shortens lifespan and decreases quality of life, but also consumes healthcare budgets, with global costs approaching £825 billion per year (2016). During the early stages of T2DM, peripheral (and possibly central (Kullmann *et al*. 2016)) insulin resistance is compensated by an increase in insulin secretion from the beta cell mass, maintaining glucose homeostasis (Samuel & Shulman 2012). However, at a poorly defined point in time, beta cells are pushed over the functional precipice, resulting in relative insulin insufficiency and a large ramp-up in circulating glucose concentration (Halban *et al*. 2014). Together with dyslipidaemia, hyperglycaemia drives the wide-ranging sequelae of T2DM including retinopathy, neuropathy, vasculopathy, renal and cardiovascular disease and increased risk of developing cancer (Forbes & Cooper 2013). In addition to diet and exercise, T2DM treatment relies on the lifelong use of oral anti-diabetic agents, which boost insulin release, improve insulin action or increase renal glucose excretion (Fowler 2007). However, a significant number of patients will eventually transition to exogenous insulin injection, probably reflecting the loss of any residual beta cell function (Home *et al*. 2014).

While current T2DM treatment aims to preserve endogenous insulin release for as long as possible, prevention of beta cell failure remains an important goal. The mechanisms through which single beta cells fail are well defined, at least in rodents, and encompass loss of glucose responsiveness, de-differentiation, ER stress and apoptosis, all probably secondary to a combination of glucolipotoxicity and cytotoxicity (Porte & Kahn 2001, Prentki & Nolan 2006). Maintenance of insulin release during T2DM therefore requires better understanding of the mechanisms underlying beta cell proliferation and function in adults, with the hope that these can be harnessed or maintained to restore insulin secretion. Recent studies have opened up new avenues in our investigation of beta cell failure and insulin secretion, by showing the existence of subtle molecular and cellular differences between beta cells which give rise to proliferative and functional subpopulations (Gutierrez *et al*. 2017). This ‘heterogeneity’ or ‘diversity’ is driven partly by the organisation of beta cells into islet microorgans (Benninger & Piston 2014) and is unlikely to be properly appreciated by current models of beta cell failure. Importantly, beta cell subpopulations may contribute to development and proliferation of other beta cells, as well as play an exaggerated role in insulin secretion (Roscioni *et al*. 2016, Avrahami *et al*. 2017, Carrano *et al*. 2017). Thus, beta cell heterogeneity or diversity should be an important consideration when attempting to understand T2DM pathogenesis, since subpopulations may be differentially targeted by insults or treatments. Conversely, islet-like structures engineered from induced pluripotent stem cells for transplantation, and even genome editing, may need to take into account beta cell heterogeneity.

The present review will discuss the influence of beta cell heterogeneity on islet function and insulin release, and its relevance for T2DM. Particular attention will be paid to recent single-cell studies describing some of the molecular and cellular underpinnings of beta cell heterogeneity. We will also focus on the role of the islet context in giving rise to beta cell heterogeneity by discussing *in situ* and *in vivo* imaging studies. Finally, new technologies available for the precise interrogation of beta cell heterogeneity will be described, before highlighting future challenges for the field, including translation of results to the clinic.

## Stimulus–secretion coupling in single beta cells

Beta cells are well adapted as glucose sensors. Due to expression of low-affinity glucose transporters (GLUT1 in humans, GLUT2 in rodents) and glucokinase (German 1993, De Vos *et al*. 1995), beta cells only respond to elevated glucose, avoiding the inappropriate and damaging release of insulin. Following transport into the cytosol, the sugar stimulates oxidative and glycolytic metabolism, leading to ATP generation in the mitochondria at the expense of other pathways (e.g. pentose phosphate shunt). The ensuing increase in cytosolic ATP/ADP ratio closes ATP-sensitive K^+^ (K_ATP_) channels, reducing K^+^ efflux through the Kir6.2 pore and depolarising the membrane (Ashcroft *et al*. 1984). Together with Na^+^ conductance, this drives action potential firing and the opening of voltage-dependent Ca^2+^ channels (VDCC): primarily L-type in rodents but T-, P/Q- and L-type in humans (Rorsman & Braun 2013, Rutter & Hodson 2013). The increased cytosolic Ca^2+^ fluxes interact with small N-ethylmaleimide-sensitive factor receptor proteins such as vesicle-associated membrane protein, synaptosomal-associated protein of 25 kDa (SNAP25) and syntaxin (Wheeler *et al*. 1996, Kwan & Gaisano 2009), stimulating insulin granule translocation to the membrane, granule fusion and dissolution of insulin crystals into the circulation. Insulin secretion is further assisted by ‘amplifying’ pathways (e.g. cAMP), which are K_ATP_ channel independent but beta cell metabolism-dependent (Henquin 2000). Indeed, molecules such as hormones, neurotransmitters, nucleotides, amino acids and lipids are all able to strongly influence insulin secretion by either acting as nutrients or through interactions with receptors/ion channels. Perhaps the best characterised signals are derived from the incretin hormones glucagon-like peptide-1 (GLP-1) and glucose-dependent insulinotropic peptide (GIP) released from the enteroendocrine L-cells and K-cells, respectively, following food intake and bile acid transit (Diakogiannaki *et al*. 2012, Parker *et al*. 2012). GIP and GLP-1 bind to their cognate G-protein-coupled receptors (GPCRs) on beta cells, stimulating adenylate cyclase activity and cAMP generation, activation of PKA and Epac, as well as ERK and beta arrestins (Baggio & Drucker 2007, Leech *et al*. 2011, Campbell & Drucker 2013). Through interactions with VDCC and the exocytotic machinery (MacDonald *et al*. 2002, Gromada *et al*. 2004, Leech *et al*. 2011), GIP and GLP-1 boost insulin secretion (so-called ‘incretin effect’), largely accounting for the extra portion of the hormone secreted in response to oral vs intravenous glucose (Nauck *et al*. 1986, Nauck & Meier 2016). Since GIP receptors are downregulated/desensitised during obesity/T2DM (Lynn *et al*. 2001, Meier & Nauck 2010), stabilised variants of GLP-1 have served as a template for production of incretin mimetics. For an in-depth review of stimulus–secretion coupling in the beta cell, the reader is referred to (Rutter *et al*. 2015).

## Single-cell studies of beta cell heterogeneity

The concept of heterogeneity is not new. Differences in insulin release (Salomon & Meda 1986), glucose metabolism (Kiekens *et al*. 1992), glucokinase (Jetton & Magnuson 1992), insulin expression (Schuit *et al*. 1988), membrane potential (Dean & Matthews 1968) and Ca^2+^ (Zhang *et al*. 2003, Kenty & Melton 2015), cAMP (Dyachok *et al*. 2006) and NAD(P)H (Piston *et al*. 1999) signals have all been described in beta cells. However, it is only over the past decade that advances in imaging, genomics and proteomics have allowed a more detailed snapshot of the molecular and cellular drivers of beta cell heterogeneity. A spate of single-cell screening studies (See 1 in Table 1) in mouse and human islets have segregated out a number of beta cell subpopulations according to their transcriptomic profile, for example, due to differing abundances of genes for ER/oxidative stress (Baron *et al*. 2016, Muraro *et al*. 2016), and in the process have provided useful beta cell ‘atlases’ (see Supplementary methods for a summary of single-cell screening methodology, as well as the section on supplementary data given at the end of this article). However, relatively few studies have investigated subpopulation plasticity and its potential to influence disease or islet function. Using marker analysis coupled with single-cell RNA sequencing (scRNA-seq), Dorrell *et al.* were able to show the existence of four distinct human beta cell subpopulations (β1–4), based upon differing expression of ST8SIA1 and CD9. Notably, the ST8SIA1-positive β3 and β4 populations exhibited lower insulin release during T2DM (Dorrell *et al*. 2016). At the protein level, high-throughput analyses of human beta cells immunostained with metal-labelled antibodies and subjected to time-of-flight mass cytometry (CyTOF) (See 2 in Table 1) showed three states: C1, C2 and C3 (Wang *et al*. 2016*a*). The C1 state – shown to be PDX1^high^/Ins^high^ and poorly proliferative – was plastic depending on age and obesity, and increased in incidence with time (Wang *et al*. 2016*a*). This suggests that, during ageing and metabolic stress, there is a shift from a proliferative to a more mature beta cell phenotype, which may partly explain the limited capacity to regenerate beta cells in the adult, as well as their eventual failure under chronic metabolic burden.

**Table 1:** Summary of technologies available for interrogating beta cell heterogeneity.

Technology	Advantages	Disadvantages	Reference
RNA-seq (1)			Ozsolak & Milos (2011)
	• Transcriptome-wide coverage • Sensitive – requires ng of DNA	• Islet dissociation required • Cells with upregulated stress pathways generally excluded from analysis • Still expensive • Biological and technical variability due to (1) loss of transcripts during RNA isolation or (2) transcript coverage	
CyTOF (2)			Proserpio & Lönnberg (2016)
	• Parallel screening of up to 40 markers • Three orders of magnitude between detection channels • Excellent discrimination of negative and positive populations • Good discrimination of high-low-negative populations	• Islet dissociation required • Expensive machinery required • Antibody panels available, but limited selection for metabolism • Antibodies need to be labelled in house if not available • General lack of reliable antibodies for some beta cell proteins/markers	
Optogenetics (3)			Johnston *et al*. (2016)
	• Conditionally targeted • Well characterised • Spectral variants • Stimulatory or inhibitory variants	• Presence of a non-mammalian channel/pump may affect cell function • Protein overexpression may interfere with cell function • Limited use in human tissue	
Photopharmacology (4)			Broichhagen *et al*. (2015*c*)
	• Usually based on known drugs/molecules • No need for genetic introduction • Exogenously applied • Can be spectrally-tuned • Useful in human tissue • Not conditional	• Non-binary response (i.e. generally some activity) • Can undergo metabolism depending on molecule • Subject to normal pharmacokinetics/pharmacodynamics • UV light is required for photoswitching of the majority of available compounds	
Tethered pharmacology (5)			Podewin *et al*. (2018)
	• Usually based on known drugs/molecules • Conditionally targeted using enzyme self-labels or engineered cysteine residues. • Can be combined with photopharmacology to make tethered photoswitches	• Use in human and mouse primary tissue requires viral vectors • Protein overexpression may interfere with cell function	
Single-cell metabolomics (6)			Aerts *et al*. (2014), Ibanez *et al*. (2013)
	• Dynamic snapshot of cell metabolism • Can be performed in the intact tissue setting	• Slow throughput compared to CyTOF and RNA-seq • Sensitivity still relatively poor compared to conventional metabolomics	
Transcriptome *in vivo* analysis (7)			Lovatt *et al*. (2014)
	• Can be performed in the intact tissue	• Requires cell-penetrating peptides to introduce the TIVA-tag • Slow throughput	
*In vivo* imaging (8)			Speier *et al*. (2008*b*)
	• Allows investigation of beta cell function with preserved blood and neural supplies	• Technically challenging • Needs expensive microscopy equipment • Slow throughput	

The numbers in brackets indicate position in the main body of the manuscript.

Analysis of cell surface markers revealed that the Wnt/planar polarity effector, Fltp or Flattop, demarcates proliferative and mature beta cells (Bader *et al*. 2016). Fltp^+^ beta cells are more mature, with improved insulin secretion and mitochondrial function, whereas the Fltp^−^ beta cell subpopulation expands during metabolic stress, displaying increased proliferation. Thus, an increase in the ratio of Fltp^−^/Fltp^+^ cells may be expected to promote/maintain beta cell mass during T2DM, but would be expected to compromise insulin release due to their relatively poor glucose responsiveness. Along similar lines, Van der Meulen *et al.* identified a rare (~2% proportion) subpopulation of beta cells, characterised by the absence of urocortin 3 (Ucn3) expression, which represent an intermediate stage during the transdifferentiation of alpha to beta cells, thus acting as a ‘neogenic niche’ (van der Meulen *et al*. 2017). Ucn3^−^ cells were metabolically naive (i.e. low glucokinase and oxidative phosphorylation) and were unable to properly sense glucose or support Ca^2+^ fluxes in response to glucose (van der Meulen *et al*. 2017), supporting previous observations that functional beta cell maturation is associated with increased Ucn3 expression (Blum *et al*. 2012). See Table 2 for a summary of selected beta cell subpopulations involved in islet plasticity and insulin release. The reader is referred to (Roscioni *et al*. 2016, Avrahami *et al*. 2017, Carrano *et al*. 2017, Gutierrez *et al*. 2017) for more comprehensive reviews of subpopulations identified to date.

**Table 2:** Selected beta cell subpopulations involved in islet plasticity and insulin release.

Subpopulation	Features	Plasticity	Reference
ST8SIA1^−^	GLUT2+++	↓ T2DM	Dorrell *et al*. (2016)
	Insulin+++		
	Maturity+++		
			
ST8SIA1^+^	GLUT2+	↑ T2DM	Dorrell *et al*. (2016)
	Insulin+		
	Maturity+		
			
C1	Insulin+++	↑ Ageing	Wang *et al*. (2016*a*)
	Maturity+++	↓ Obesity	
			
Fltp^+^	Metabolism+++	↓ Metabolic stress	Bader *et al*. (2016)
	Insulin+++		
	Maturity+++	↓ Metabolic stress	
	Proliferation+		
			
Fltp^−^	Metabolism+	↑ Metabolic stress	Bader *et al*. (2016)
	Insulin+		
	Maturity+	↑ Metabolic stress	
	Proliferation+++		
			
Ucn3^−^	Metabolism+	↑ Metabolic stress	van der Meulen *et al*. (2017)
	Ca^2+^+		
	Maturity+		
	Transdifferentiation+++		
			
Hub (eNpHR3.0)	Metabolism+++	↓ Metabolic stress	Johnston *et al*. (2016)
	Ca^2+^+++		
	Insulin++		
	Maturity++		
			
ChR2	Metabolism+++	↓ Metabolic stress	Westacott *et al*. (2017*b*)
	Ca^2+^+++		

Together, high-throughput scRNA-seq, CyTOF or reporter studies have provided unexpected insights into beta cell heterogeneity, identifying a number of subpopulations that display high degree of plasticity. The binary nature of these subpopulations appears to be a common trait: subgroups with two broad characteristics predominate during metabolic stress and ageing. Beta cells with decreased maturity, glucose sensing, metabolism and insulin secretion increase in proportion during metabolic stress, which may reduce islet function at the expense of maintaining beta cell renewal. Nevertheless, this may be balanced by expansion of beta cells with increased maturity and insulin expression, but poorer proliferative capacity. Whether these phenotypic shifts are interdependent is not known, but this raises the tantalising prospect that the islet is geared toward preserving heterogeneity and that eventual loss of this (i.e. as a subpopulation begins to predominate) is a key trigger for insulin secretory failure.

There are a number of caveats that need to be carefully considered when interpreting transcriptomic and biomarker-based studies. RNA-seq is limited in its coverage of the genome, with ~10% genes being missed even at high read numbers (Anisimova *et al*. 2015). While CyTOF resolution is excellent compared to conventional fluorescence-activated cell sorting (FACS) (Giesen *et al*. 2014), the poor availability of metal-labelled antibodies for many markers means that screening is still limited/partially biased, although this is expected to improve with labelling kits. In marker-based studies, reporter constructs may not faithfully report protein expression, and in many cases, display limited changes in expression vs the protein under examination. Furthermore, how the subpopulations described to date co-exist together in the islet is not well understood, and this may be complicated by the relative dynamics of subpopulation plasticity. So far, most studies report results obtained at a specific time point (e.g. neonatal, postnatal and adult), without taking into account the changes that may appear in-between the time of assessment. Lastly, following dissociation from the islet, beta cells may lose many characteristics endowed through cell–cell contacts/signalling, certain stress pathways may be upregulated and a study may be skewed toward subpopulations that are more robust (*hint:* some are fragile, see below).

## Functional beta cell heterogeneity in the intact islet

In response to glucose, beta cells display intense Ca^2+^ oscillations, which are not completely synchronous but well-coordinated throughout the syncytium (Benninger *et al*. 2008, Stozer *et al*. 2013). By contrast, beta cells cultured in two dimensions mount much more stochastic responses to secretagogues, release less insulin *per capita* and possess decreased insulin biosynthetic capacity (Lernmark 1974, Hodson *et al*. 2013). This diminished functionality develops as a result of the loss of three-dimensional electrical communications through gap junctions following islet dissociation. Indeed, gap junctions couple on average 6–7 neighbouring beta cells, with a speed of electrical exchanges of ~13.5 ms, commensurate with the transmission of Ca^2+^ signals (~80 µm/s) (Aslanidi *et al*. 2001, Benninger *et al*. 2008, Zhang *et al*. 2008, Stozer *et al*. 2013). Studies using islets from mice globally or conditionally deleted for the gap junction protein connexin 36 (Cx36; encoded by *Gjd2*) – the major isoform expressed in beta cells (Serre-Beinier *et al*. 2000) – showed loss of coordinated responses to glucose, which resembled those observed in dissociated beta cells (Ravier *et al*. 2005, Head *et al*. 2012). Other plausible mechanisms for beta cell–beta cell communications include diffusion of chemical messengers (i.e. paracrine signalling) (Squires *et al*. 2002, Yang *et al*. 2011), contact-dependent signalling (i.e. ephrin) (Konstantinova *et al*. 2007) and ciliary (Gerdes *et al*. 2014) signalling, although the contribution of these slower communication modalities to islet function is less well characterised (reviewed in Rutter & Hodson 2014).

While human beta cells respond to glucose more stochastically (Martin & Soria 1996, Cabrera *et al*. 2006), regional coordination is detected between defined clusters (Cabrera *et al*. 2006, Westacott *et al*. 2017*a*), probably reflecting the differing architecture of islets in humans, where beta cell–beta cell interactions and gap junction signalling are constrained by an extra folding step during development (Bosco *et al*. 2010, Steiner *et al*. 2010). Human beta cells appear to be more dependent on the gut-derived peptide GLP-1 for coordinated responses. Incretin application induces large and synchronous Ca^2+^ rises that are lost following shRNA-mediated knockdown of *GJD2* or islet dissociation (Hodson *et al*. 2013). We speculate that the greater dependency on incretin input for coordination between human beta cells may reflect a mechanism to tightly entrain insulin release to mealtime, as GLP-1 is released from enteroendocrine L-cells following food ingestion (Diakogiannaki *et al*. 2012). By contrast, rodents graze during the dark phase, and in this scenario, glucose may provide adequate entrainment of insulin release with food intake. Further studies should look into the role of the enteroinsular axis in beta cell connectivity both in humans and rodents.

**Figure 1:**
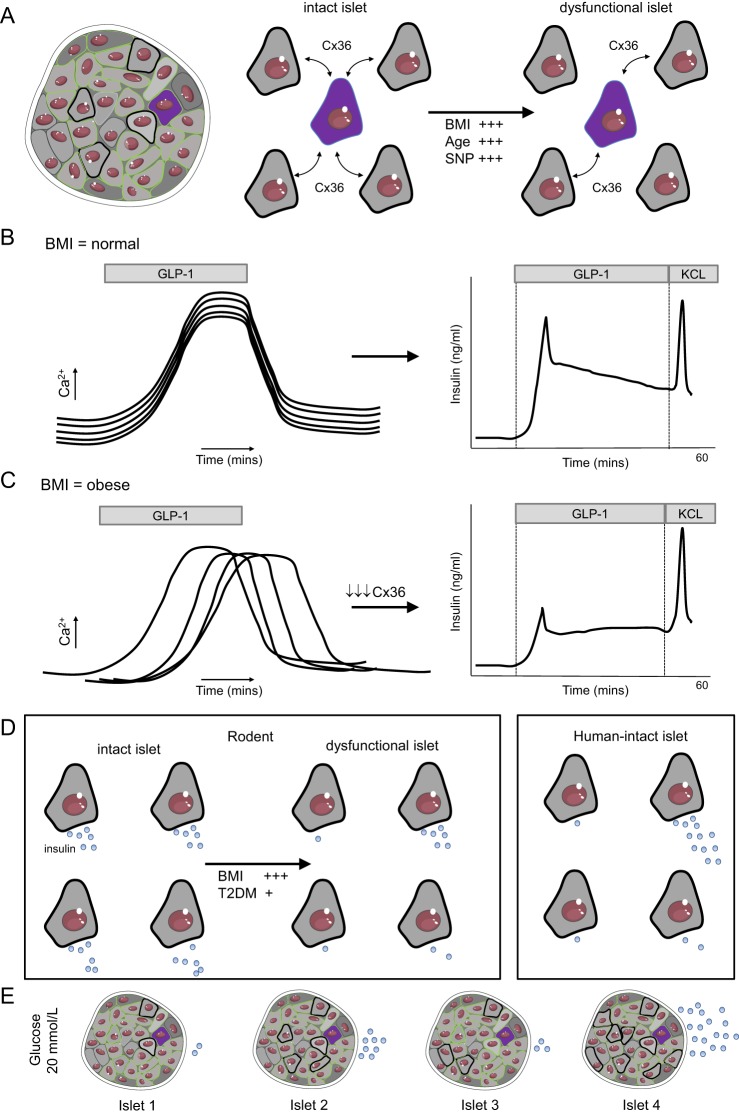
Beta cell communication and insulin release: (A) Gap junctions comprising connexin 36 allow beta cells to coordinate their activities within the intact islet. Gap junction signalling is reduced during obesity and ageing, as well as in individuals harbouring risk alleles for T2DM (SNP). (B) Beta cells in human islet mount coordinated responses to incretins such as glucagon-like peptide 1 (GLP-1), facilitating insulin release. (C) During obesity, a reduction in gap junction signalling leads to loss of coordinated beta cell activity, impairing GLP-1-stimulated insulin secretion. (D) Insulin secretion is polarised toward the vasculature and in rodent models of obesity and T2DM, a reduction in the number of actively secreting beta cells is detected. In humans, some beta cells and beta cell clusters contribute to insulin secretion more than others. (E) Islets display a large functional reserve, with only a handful of first responders supporting glucose-stimulated insulin secretion. Figures were adapted from Servier Medical Art under a CC-BY3.0 licence (https://creativecommons.org/licenses/by/3.0/).

Coordinated beta cell–beta cell behaviour appears to be important for normal islet function and insulin release. Mice lacking *Gjd2*/Cx36 show lowered indices of coordinated beta cell Ca^2+^ activity and loss of pulsatile insulin secretion both *in vitro* (Ravier *et al*. 2005) and *in vivo* (Head *et al*. 2012), leading to glucose intolerance despite preserved steady-state insulin release (Ravier *et al*. 2005, Head *et al*. 2012). Similarly, beta cell–beta cell coordination may be relevant for islet failure during T2DM. The beta cell population in islets from genetically obese *ob/ob* mice displays poorly organised responses to glucose with loss of pulsatile insulin release (Ravier *et al*. 2002), and Cx36 expression and gap junction coupling strength between beta cells is markedly reduced by exposure to a pro-inflammatory cytokine cocktail (Farnsworth *et al*. 2015, Johnston *et al*. 2016). In human islets, coordinated beta cell responses to GLP-1 are inversely correlated with donor BMI and are lost following incubation with excess fatty acids, possibly due to a reduction in Cx36 expression (Hodson *et al*. 2013) (Fig. 1A, B and C). A decline in beta cell coordination and gap junction coupling was also associated with defective insulin secretory dynamics in islets from older donors (Westacott *et al*. 2017*a*). Lastly, knockdown of *ADCY5* and *Tcf7l2*, genes identified by GWAS as potentially harbouring single nucleotide polymorphisms (SNPs) increasing T2DM risk, led to marked decreases in beta cell–beta cell coordination (Hodson *et al*. 2014, Mitchell *et al*. 2014), as well as impaired glucose- and GLP-1-stimulated insulin secretion. Thus, loss of proper gap junction coupling and coordinated beta cell responses to secretagogues, may lead to impaired insulin release under conditions associated with T2DM (i.e. obesity, inflammation and gene silencing). For a comprehensive review of heterogeneity in the islet, see (Benninger & Piston 2014).

## Beta cell heterogeneity and insulin secretion

Differences in insulin secretion between individual beta cells were originally described using haemolytic plaque assay, including plasticity in response to pregnancy (Salomon & Meda 1986, Hiriart & Ramirez-Medeles 1991, Maedler *et al*. 2006). Subsequent studies using total internal refraction microscopy (TIRF-M) reported heterogeneity in secretory vesicle behaviour in rodent and human beta cells, including kiss-and-run exocytosis where fusion with the membrane occurs transiently, facilitating vesicle reuse (Tsuboi & Rutter 2003, MacDonald *et al*. 2006, Rutter & Hill 2006, Hanna *et al*. 2009), as well as differences in submembrane granule turnover and motility depending on stimulus and granule age (Hoboth *et al*. 2015, Bruning *et al*. 2017). However, TIRF-M only encompasses a dozen or so beta cells (Li *et al*. 2013), limiting the study of secretory heterogeneity across the entire islet. Nearly 20 years later, the advent of two-photon imaging enabled the tracking of the granule fusion with the beta cell membrane at nanometer axial resolution. Pioneering studies by Takahashi *et al.* deployed two-photon extracellular polar tracer imaging-based quantification (TEPIQ) to report uptake of the polar tracer, sulforhodamine B, into granules following fusion with the membrane, allowing exocytosis to be tracked via the appearance of fluorescent spots (Takahashi *et al*. 2002). Almost 60% of the beta cells were found to be involved in exocytosis, with the majority of events occurring toward the interstitial or non-vascular compartment. Interestingly, kiss-and-run exocytosis in the intact islet setting is an exceedingly rare event, suggesting heterogeneity is strongly shaped by cell–cell interactions (and possibly extracellular matrix content) (Takahashi *et al*. 2002, Ma *et al*. 2004). However, some polarity in insulin release was detected in intact islets, with exocytosis preferentially toward the vessels surrounded by beta cell rosettes (Takahashi *et al*. 2002). Similar TEPIQ studies showed that glucose recruits single cells throughout the islet to exocytosis, increases the number of fusion events per beta cell and induces coordinated oscillatory exocytotic activity, reminiscent of Ca^2+^ signals (Low *et al*. 2013) (Fig. 1D). The same group showed that, when viewed in three dimensions, insulin secretion is asymmetric and targeted toward the vasculature (Fig. 1D), probably due to enrichment of liprin, piccolo and Rab2-interacting molecule at the vascular face (Low *et al*. 2014). Notably, beta cells in islets from glucose intolerant *db/db* mice showed some loss of insulin secretory heterogeneity, with 73% of cells becoming refractory to stimulation (Fig. 1D), although polarisation toward the vasculature was not studied (Do *et al*. 2014). In human islets, granule release was shown to be pulsatile/oscillatory, with coordination detected only between beta cell clusters. The difference in coordination reflects the compartmentalisation of the Ca^2+^ response in this species, which could mirror the arrangement of beta cells vs alpha cells (Cabrera *et al*. 2006, Almaca *et al*. 2015). In contrast to the mouse, a minority of beta cells contribute the majority of insulin secretion in humans, as assessed using haemolytic plaque assays (Wojtusciszyn *et al*. 2008) (Fig. 1D), although this may imply a prolonged secretion where secretory events tend to become more localised (Almaca *et al*. 2015). How heterogeneity in insulin secretion changes during T2DM is not well understood, probably due to difficulties in obtaining fresh tissue from donors. However, studies using matrix-assisted laser desorption ionisation (MALDI) imaging in fixed tissue, together with* in vitro* experiments, demonstrated that stearoylcarnitine accumulated in beta cells to arrest insulin synthesis, while acetylcarnitine and N-acyl taurines increased insulin secretion to induce beta cell failure (Aichler *et al*. 2017).

**Figure 2:**
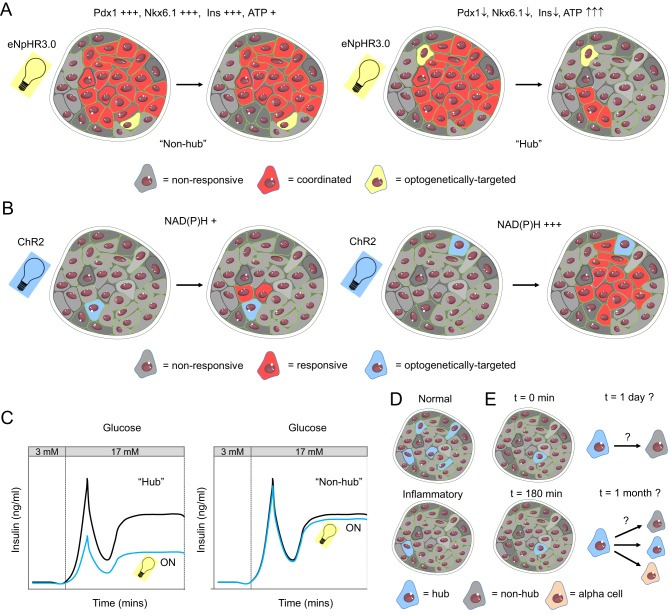
Beta cell heterogeneity and islet function. (A) Optogenetic silencing of less mature and metabolically‑adapted beta cells, termed hubs, leads to a loss of coordinated population activity (eNpHR3.0; halorhodopsin3.0, a yellow light-activated Cl^−^ pump). (B) Optogenetic activation of cells with high but not low NAD(P)H levels leads to activation across the islet (ChR2, channel rhodopsin 2, a blue light-activated Na^+^ channel). (C) Insulin secretion is impaired following silencing of hubs, but not other single beta cells. (D) Hubs are targeted by cytokines to mimic the pro-inflammatory milieu associated with T2DM. (E) Hubs are stable for a few hours, but their fate is not known over longer time periods. Possibilities include: (1) remaining a hub; (2) becoming a non-hub beta cell or (3) transdifferentiation into another cell type. Figures were adapted from Servier Medical Art under a CC-BY3.0 licence (https://creativecommons.org/licenses/by/3.0/).

By contrast to *in vitro* experiments, technically demanding live imaging of the pancreatic surface in anaesthetised animals revealed that only a fraction of islets responded to acute glucose administration (Fig. 1E). This was characterised by rapid dispossession of insulin, transgenically marked using C-peptide–bearing superfolder GFP (Zhu *et al*. 2016). While the contribution of individual beta cells was likely not determined at the resolutions employed in these studies, it is nonetheless intriguing that most islets are unresponsive, suggestive of a functional reserve and tertiary level of heterogeneity (Zhu *et al*. 2016). Thus, significant heterogeneity in insulin secretion exists *in vitro*, with recruitment, coordination and polarisation toward the vasculature, with species differences appearing to reflect Ca^2+^ patterning. Further studies are required to understand how this is sculpted *in vivo*, where neural and vascular supplies may also influence beta cell function, as well as the fate of secreted insulin depending on the metabolic status (Michau *et al*. 2015).

## Functionally interrogating beta cell heterogeneity *in situ*

Although coordinated, Ca^2+^ responses to glucose vary in space and time within the beta cell compartment. Some islet sub-regions respond early to stimulus (Stozer *et al*. 2013), with Ca^2+^ responses propagating as gap junction-dependent waves across the islet (Benninger *et al*. 2008, 2014), indicating a presence of so-called pacemakers (Ämmälä *et al*. 1991, Squires *et al*. 2002, Benninger *et al*. 2014). Thus, beta cell–beta cell communication and Ca^2+^ signalling *in situ* are clearly heterogeneous. Optogenetics (See 3 in Table 1) allied to high-speed imaging has recently opened up the possibility to precisely interrogate the influence of beta cell heterogeneity directly in the intact islet, where endocrine cell interactions critical for proper insulin release are preserved. Studies by Reinbothe *et al.* and Kushibiki *et al.* employed channel rhodopsin 2 (ChR2), a light-activated Na^+^ channel, to optically control insulin release from beta cells *in vitro*, as well as *in vivo* in mice rendered diabetic with streptozotocin (Reinbothe *et al*. 2014, Kushibiki *et al*. 2015). Subsequent studies by our lab using the yellow/orange light-activated Cl^−^ pump halorhodopsin 3.0 (NpHR3.0) to pinpoint silence cell activity revealed that a minority of beta cells, termed hubs, are tasked with orchestrating islet-wide Ca^2+^ and insulin secretory responses to glucose (Johnston *et al*. 2016) (Fig. 2A and C). These beta cells constitute ~1–10% of the population at the islet surface, display activity profiles that precede and lead those of the rest of the population (i.e. pacemaker-like) and tend to host the majority of coordinated activity (*think:* network servers or major airports). To support their activity, hubs possess high levels of glucokinase and highly hyperpolarised mitochondria, indicative of increased ATP synthase activity and ATP generation. Unexpectedly, this was associated with lowered but not absent Pdx1 and Nkx6.1 and reduced insulin expression, resembling cells identified using RNA-seq (GK^high^/Pdx1^low^/Nkx6.1^low^) (Xin *et al*. 2016) and gene reporters (Pdx1^+^/insulin^low^) (Szabat *et al*. 2009), and suggestive of a relatively immature phenotype.

How does metabolic adaptation in hubs fit with the apparent loss of beta cell identity, given that the later supposedly endows the former? Maturity, as defined by expression of transcription factors such as Nkx6.1, Pdx1 and MafA may not always be obligatory for glucose metabolism. For example, NAD(P)H responses in beta cells deficient in MafA are almost normal (Hang *et al*. 2014) and deletion of a single *Pdx1* allele in beta cells increased apoptosis, but did not affect Ca^2+^ fluxes, Ca^2+^ conductance, glucose sensing or insulin secretion *in vitro* (Johnson *et al*. 2003). In addition, hubs may represent an immature and proliferative subpopulation in adults, similar to Ucn3^−^ cells (van der Meulen *et al*. 2017), although differentiated by their normal glucose responsiveness, heightened mitochondrial metabolism and improved Ca^2+^ fluxes. Indeed, replicating beta cells double RNA abundance of the majority of genes, except for those involved in key beta cell functions such as secretion (Klochendler *et al*. 2016). During postnatal development, immature proliferative cells possess high expression levels of amino acid metabolism and mitochondrial genes (Zeng *et al*. 2017). Nonetheless, hubs were susceptible to failure following introduction of a pro-inflammatory milieu, which may reflect a relative lack of Pdx1 and SERCA2, the former interacting with the latter to maintain organellar Ca^2+^ homeostasis and protect against ER stress (Sachdeva *et al*. 2009, Johnson *et al*. 2014).

Using ChR2, Westcott *et al.* activated single beta cells at random and calculated the proportion of the islet showing corresponding Ca^2+^ elevations (Westacott *et al*. 2017*b*). While these elevations were restricted to closest neighbours in most experiments, in ~5% of trials Ca^2+^ spread over large islet regions. Suggesting metabolic adaptation in these cases, the Ca^2+^ elevation was associated with cells/sub-regions displaying the highest NAD(P)H responses (Westacott *et al*. 2017*b*; Fig. 2B). However, the same authors showed that areas with low NAD(P)H and high Ca^2+^ oscillation frequency were associated with the origin of Ca^2+^ wave initiation. Whether these optogenetically defined beta cell subpopulations are distinct, or are overlapping but arise as a consequence of the wave propagation/correlation analysis used, needs to be further investigated. These studies together show that beta cell subpopulations *in situ* can play a disproportionate role in dictating islet responses to glucose, that this may be due to alterations in metabolism, and that these cells may fail in response to diabetes-like insults (Fig. 2A-E). It will be interesting to determine if these subpopulations have a similar protein barcode to hubs or other identified beta cell subpopulations that display metabolic adaptation.

Whether or not optogenetically defined beta cell subpopulations are stable or dynamic is difficult to assess due to restrictions on recording time *in vitro* (a few hours at most; Fig. 2E). Modelling studies have shown that pacemaker regions may stem from areas of highest excitability (Benninger *et al*. 2014), and this may change depending on ion channel expression levels (turnover times for Kir6.1/SUR1 complexes are estimated to be ~2.2 h) (Crane & Aguilar-Bryan 2004) or proximity to non-beta cells (such as delta cells, shown to convey beta cell-driven alpha cell suppression) (Briant *et al*. 2017)). Moreover, how activity of single beta cells and small sub-regions can lead to Ca^2+^ signal propagation across large regions of the islet is not known. Presumably, gap junctions play a role, since their knockdown decreases the distance over which signals travel (Johnston *et al*. 2016) and disrupts beta cell–beta cell coordination (Ravier *et al*. 2005, Head *et al*. 2012). Other explanations may lie outside of the field of view, since most experiments only consider the top couple of islet layers. While 3D imaging of mouse islets is challenging due to the highly scattering nature of endocrine cells densely packed with secretory granules, studies in the more transparent zebrafish model using light sheet microscopy are likely to be more informative (Singh *et al*. 2017).

As a technology for interrogation of beta cell function, optogenetics is not without drawbacks. First, high (and potentially phototoxic) laser powers are required to activate ChR2 and especially eNpHR3.0, and constant light exposure can ground-deplete their activity cycle (i.e. render them refractory). Second, Cl^−^ and H^+^ pumps required for cell silencing can generate profound hyperpolarisation, similar to full K_ATP_ channel opening, with yet unknown consequences on long-term beta cell function. The mechanisms occurring in beta cells likely involve transcriptional changes, given the recently reported interplay between Ca^2+^ influx and gene networks (Stancill *et al*. 2017). Third, ChR2 and eNpHR3.0 significantly improved glucose tolerance *in vivo* without illumination (Reinbothe *et al*. 2014, Johnston *et al*. 2016), an effect that cannot be explained by their activity, as no illumination was present. Lastly, all optogenes introduce an exogenous photocurrent, and while no issues have been reported for ChR2, an increase in beta cell excitability was reported following inactivation of eNpHR3.0 (Johnston *et al*. 2016), probably due to a collapse in the Cl^−^ gradient and depolarisation following a shift in the Cl^−^ reversal potential (Raimondo *et al*. 2012). Thus, as for RNA-seq, CyTOF and other techniques used in the study of beta cell heterogeneity, careful consideration of the datasets is required.

## Beta cell heterogeneity – more questions raised than answered?

Beta cell heterogeneity is highly plastic in response to a pro-diabetic milieu. Thus, ST8SIA1^+^ beta cells increase in proportion in islets from T2DM donors (Dorrell *et al*. 2016), similar to Fltp^−^ beta cells that expand in number following metabolic stress (Bader *et al*. 2016). Hubs, as well as gap junction signalling, fail in the face of inflammation (Farnsworth *et al*. 2015, Johnston *et al*. 2016), while beta cell ion channel number is altered during T2DM (Dorrell *et al*. 2016, Wang *et al*. 2016*b*), and exocytosis is restricted to only a few beta cells in islets from obese *db/db* mice (Low *et al*. 2014). However, the contribution of each event to T2DM and their inter-relations remain unclear. Are alterations in gap junction signalling preceded by subpopulation shifts to a more immature phenotype? If so, how can this be reconciled with the known molecular mechanisms underlying decreased gap junction coupling (i.e. protein kinase C delta) or evident heterogeneity in gap junction coupling strength (Farnsworth *et al*. 2015)? What is the overlap between the various subpopulations, given that mature Fltp^+^ and ST8SIA^−^ cells both constitute ~80% of the beta cell population? What are the relative dynamics of subpopulation shifts (e.g. from ST8SIA^−^ -> ST8SIA1^+^) and how does this relate to changes in function and insulin release? Are these shifts permanent or can they be reversed following treatment of T2DM or remission in mouse models? Key to answering some of these questions will be technologies that allow heterogeneity to be longitudinally tracked with high temporal resolution.

## New tools for understanding beta cell heterogeneity

The majority of recent understanding concerning beta cell heterogeneity has been derived from ‘omics’ or high-throughput approaches, such as RNA-seq and CyTOF. While these have identified subpopulations with similar characteristics in terms of maturity and insulin secretion, they have also highlighted differences in terms of the markers/genes that are differentially expressed. However, further description of heterogeneity will require the use of complementary techniques, as well as integration of the obtained datasets to increase detection sensitivity and accuracy.

### Photopharmacology

**Figure 3:**
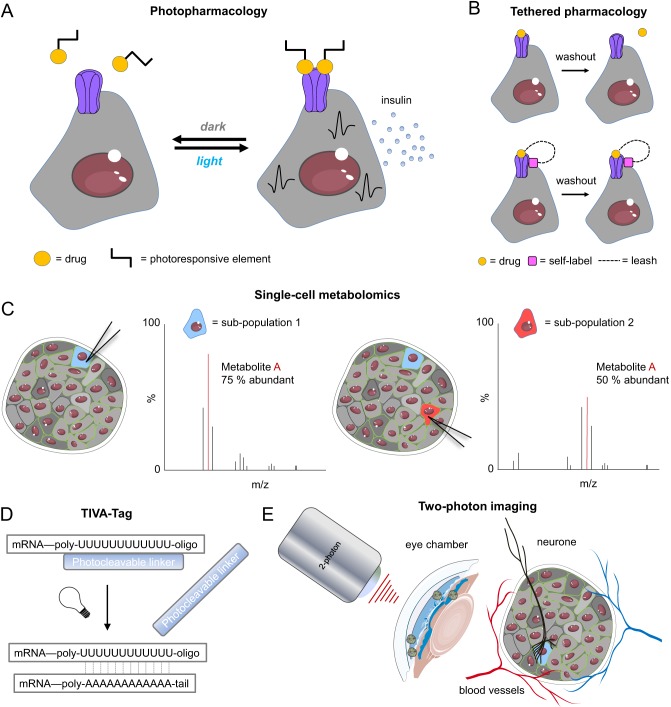
Future technologies for interrogating beta cell heterogeneity. (A) Photopharmacology uses light-activated drugs to turn receptors and ion channels into endogenous photoswitches. (B) Tethered pharmacology combines the precision of genetics with pharmacology to selectively target specific cell and receptor populations, and even organelles. (C) Single-cell metabolomics subjects cytoplasm extracted from specific cells to capillary electrophoresis-mass spectrometry, potentially delineating differences in metabolite abundances between subpopulations. (D) TIVA-tag affords optical control over mRNA capture in single cells using a photocleavable poly-U-oligo, which hybridises the corresponding poly-A-tail. (E) Two-photon imaging of islets transplanted into the anterior chamber of the eye allows beta cell heterogeneity to be visualised in a setting where vascular and neural supplies re-wire. Figures were adapted from Servier Medical Art under a CC-BY3.0 licence (https://creativecommons.org/licenses/by/3.0/).

Photopharmacology, otherwise referred to as optochemistry, describes the use of light to control the cell’s endogenous signalling apparatus/cytoskeleton (Velema *et al*. 2014). This approach relies on the synthesis of molecules whose inactive and active states can be precisely controlled by light. Central to photopharmacology, is the installation of photoresponsive elements, usually an azobenzene moiety, which undergoes isomerisation in response to illumination (a molecular motor) (Broichhagen *et al*. 2015*c*) (Fig. 3A) (See 4 in Table 1). The ensuing shift between *cis-* and *trans*-isomers subtly influences molecular motion, altering binding conformations and thus placing ion channel, receptor and enzyme function under optical control (Broichhagen *et al*. 2015*c*). A major advantage of photopharmacology over optogenetics is the lack of requirement for recombinant engineering: drugs can be exogenously applied, for example, light-activated sulfonylureas (Broichhagen *et al*. 2014, 2015*b*), incretins (Broichhagen *et al*. 2015*d*, 2016) and GPR40 agonists (Frank *et al*. 2017). Photopharmacology can serve as a tool to understand how GPCR or ion channel activation influences signalling and function in defined subpopulations, since these can feasibly be targeted with light as for hubs, with particular use in human islets. Pertinently, expression levels of *Trpm6*, and subunits for K_ATP_ and K^+^ channels all change in islets isolated from donors with T2DM (Dorrell *et al*. 2016, Wang *et al*. 2016*b*). However, there are disadvantages with photopharmacology: the lack of binary switch can complicate the optical control of cell function, UV illumination is generally required for the isomerisation step and toxicity and metabolite production need to be carefully assessed, just as for any drug, although the azobenzene itself appears to be well tolerated (Mehta *et al*. 2017).

### Tethered pharmacology

Tethered pharmacology describes the production of small-molecule and peptide agonists that are restricted to the cell surface or to a specific receptor through protein self-labels (Leippe *et al*. 2017, Shields *et al*. 2017) (Fig. 3B) (See 5 in Table 1). Generally, this relies on covalent binding through SNAP tags, but can also take the form of engineered cysteines that react with maleimides introduced into the ligand (Leippe *et al*. 2017, Shields *et al*. 2017). Thus, tethered pharmacology allows cell signalling to be conditionally targeted, with the potential to further interrogate subpopulations using light-activated congeners, Cre-recombinase-directed expression of the SNAP-tag (Broichhagen *et al*. 2015*a*, Levitz *et al*. 2017) or even SNAP GPCR fusions, as recently shown for the GLP-1R in MIN6 beta cells (Podewin *et al*. 2018).

### Single-cell metabolomics

While single-cell RNA and protein sequencing approaches provide valuable information regarding a cell’s potential, they are much less informative concerning dynamic responses to environment. By contrast, the metabolome gives a snapshot of metabolism by surveying the metabolites built up in a cell at a given time point (Johnson *et al*. 2016). Such an approach is clearly relevant for beta cells where oxidative and glycolytic metabolism underpin insulin release. However, investigating subpopulations is limited by the amount of tissue required for reliable metabolite measures, although metabolomics on FACS-enriched populations from multiple islets (e.g. of Fltp^−^, ST8SIA1^+^ or Ucn3^−^ cells) is plausible. Also, this does not take into account variability between single cells. Micro-arrays for mass spectrometry have been applied to single yeast cells, revealing co-existing subpopulations in isogenic populations (Ibanez *et al*. 2013). Along similar lines, single neurons were subjected to capillary electrophoresis-mass spectrometry and metabolomic analysis using cytoplasm extracted under direct visualisation (Fig. 3C) (See 6 in Table 1) (Aerts *et al*. 2014). While this is slow throughput, 3D holographic and tomographic laser microscopy coupled with a nanospray tip to extract picolitres of cytosol for mass spectroscopy may speed this up (Ali *et al*. 2016). Crucially, this circumvents the need to dissociate islets, meaning that metabolites can potentially be surveyed *in situ* in the whole islet.

### Transcriptome *in vivo* analysis

Endocrine cells display stable gene expression patterns in a tissue context, with this becoming pulsatile following dissociation (Featherstone *et al*. 2011). Multiple rates of transcription can also be detected, with local coordination determined by gap junction communication (Featherstone *et al*. 2016). Such influence of the 3D architecture is likely to be lost following dissociation into single cells for high-throughput analysis, meaning heterogeneity may not be fully captured. Moreover, the unique extracellular microenvironment present *in vivo* may exert another level of heterogeneity on gene expression (Michau *et al*. 2015, Arzouni *et al*. 2017). Single-cell transcriptomes can be directly assessed in the tissue context directly *in vitro* and *in vivo* using transcriptome *in vivo* analysis (TIVA)-tags, which comprise a light-activated capture oligonucleotide that binds to the mRNA polyA tail, allowing affinity purification of TIVA-mRNA hybridised cells (Fig. 3D) (See 7 in Table 1) (Lovatt *et al*. 2014). Using this technique, it was possible to show that the *in vivo* tissue microenvironment drives bimodal gene expression (27 vs 645 bimodal transcripts in single neurones from culture compared to *in vivo*, respectively) (Lovatt *et al*. 2014).

### *In vivo* imaging

Little is known about *in vivo* function of beta cell subpopulations, other than static snapshots regarding their plasticity (e.g. proliferation of Fltp^−^ cells or transdifferentiation of Ucn3^−^ cells). The ability to study islets directly *in vivo* promises to provide novel information regarding the influence of the vascular or neural supplies on beta cell heterogeneity, whether heterogeneity contributes to insulin secretion *in vivo* and the dynamics of beta cell subpopulation plasticity (e.g. is it gradual or rapid? Do cells remain in a fixed state or interchange?). To facilitate such studies, rodent and human islets can be transplanted into the anterior chamber of the eye (Speier *et al*. 2008*a*,*b*), which provides an optically translucent window into beta cell function when combined with epifluorescent, confocal or multiphoton imaging and even optogenetics (Fig. 3E) (See 8 in Table 1). Moreover, neurons and vascular supplies re-wire (Rodriguez-Diaz *et al*. 2012, Diez *et al*. 2017), affording the opportunity to investigate the influence of these on beta cell heterogeneity, and function/fate/plasticity can be followed longitudinally in the same animal (e.g. in response to high fat diet or anti-diabetic drug therapy) (Abdulreda *et al*. 2016, Paschen *et al*. 2016). However, results need to be interpreted in light of some reported immune infiltration (Mojibian *et al*. 2013) and lack of endocrine–exocrine interactions. By contrast, imaging of the pancreas via abdominal laparotomy allows investigation of endocrine–exocrine interactions and native blood and neural supplies (Nyman *et al*. 2008, 2010, Michau *et al*. 2015), but is complicated due to movement and is restricted predominantly to surface islets in the splenic region.

## Future challenges

Beta cell heterogeneity exists at many levels of islet operation, from genes > proteins > stimulus–secretion coupling > insulin release. High-throughput screening, as well as *in situ* imaging studies have provided unprecedented details regarding the molecular and cellular drivers of this heterogeneity, including subpopulations based upon their transcriptome, Ca^2+^ responses and secretory capacity. However, how heterogeneity co-exists at multiple levels is poorly described, in particular, the overlap between subpopulations. Moreover, as many subpopulations identified to date are defined by multiple markers or gene patterns, it remains difficult to functionally interrogate their contribution to islet function using conditional silencing or overexpression approaches. Technical and bioinformatic constraints also limit the sensitivity to delineate subpopulations, especially in islets from T2DM donors where despite best efforts to age, sex and BMI-match, variability can still stem from drug therapy, historic glycaemic control and timing of diagnosis. Indeed, validation of genes differentially expressed in three separate single-cell studies using islets from normal and T2DM donors, revealed overlap between 54/77 and 32/171 significantly upregulated and downregulated genes, respectively (Lawlor *et al*. 2017). Nonetheless, this in itself demonstrates the power of combining RNA-seq results to finely resolve the transcriptome. We anticipate that the ever-increasing fidelity of single-cell technologies, the combination of functional and gene expression analysis and bioinformatics integration of datasets will expand the view of beta cell heterogeneity.

Going forwards, it will be important to demonstrate the relevance of heterogeneity for individuals with T2DM. Certainly, subpopulations with proliferative capacity could be harnessed to aid beta cell regeneration, although more detailed assessment of *in situ* functional heterogeneity is required in human islets to see if this reflects the available rodent data. Conversely, screens may identify drugs that protect susceptible subpopulations, preventing beta cell failure in the first place. The generation of beta cells from iPS cells should aim to recapitulate normal heterogeneity, as changes in heterogeneity have consistently been shown to occur during T2DM, and in some cases, lead to lowered beta cell function. More broadly, the islet provides a tractable model for the study of heterogeneity in general, and results may be applicable to the study of other less accessible organs, for example, the pituitary gland where heterogeneity in at least two different axes has also been shown to be critical for proper hormone release (Sanchez-Cardenas *et al*. 2010, Hodson *et al*. 2012, Le Tissier *et al*. 2016).

## Supplementary Material

Supporting methods

## Declaration of interest

The authors declare that there is no conflict of interest that could be perceived as prejudicing the impartiality of the research reported.

## Funding

D J H was supported by a Diabetes UK R.D. Lawrence (12/0004431) Fellowship, a Wellcome Trust Institutional Support Award and MRC (MR/N00275X/1) and Diabetes UK (17/0005681) Project Grants. This project has received funding from the European Research Council (ERC) under the European Union’s Horizon 2020 research and innovation programme (Starting Grant 715884 to D J H).

## References

[bib1] NCD Risk Factor Collaboration (NCD-RisC) 2016 Worldwide trends in diabetes since 1980: a pooled analysis of 751 population-based studies with 4·4 million participants. Lancet 387 1513–1530. (10.1016/s0140-6736(16)00618-8) 27061677 PMC5081106

[bib2] Abdulreda MH Rodriguez-Diaz R Caicedo A Berggren PO 2016 Liraglutide compromises pancreatic beta cell function in a humanized mouse model. Cell Metabolism 23 541–546. (10.1016/j.cmet.2016.01.009) 26876561 PMC4785083

[bib3] Aerts JT Louis KR Crandall SR Govindaiah G Cox CL Sweedler JV 2014 Patch clamp electrophysiology and capillary electrophoresis–mass spectrometry metabolomics for single cell characterization. Analytical Chemistry 86 3203–3208. (10.1021/ac500168d) 24559180 PMC3964733

[bib4] Aichler M Borgmann D Krumsiek J Buck A MacDonald PE Fox JEM Lyon J Light PE Keipert S Jastroch M, ***et al*** 2017 N-acyl taurines and acylcarnitines cause an imbalance in insulin synthesis and secretion provoking β cell dysfunction in type 2 diabetes. Cell Metabolism 25 1334.e1334–1347.e1334. (10.1016/j.cmet.2017.04.012) 28591636

[bib5] Ali A Abouleila Y Amer S Furushima R Emara S Equis S Cotte Y Masujima T 2016 Quantitative live single-cell mass spectrometry with spatial evaluation by three-dimensional holographic and tomographic laser microscopy. Analytical Sciences 32 125–127. (10.2116/analsci.32.125) 26860553

[bib6] Almaca J Liang T Gaisano HY Nam HG Berggren PO Caicedo A 2015 Spatial and temporal coordination of insulin granule exocytosis in intact human pancreatic islets. Diabetologia 58 2810–2818. (10.1007/s00125-015-3747-9) 26376795 PMC6132229

[bib7] Ämmälä C Larsson O Berggren P-O Bokvist K Juntti-Berggren L Kindmark H Rorsman P 1991 Inositol trisphosphate-dependent periodic activation of a Ca2+-activated K+ conductance in glucose-stimulated pancreatic β-cells. Nature 353 849–852. 1719424 10.1038/353849a0

[bib8] Anisimova M García-Ortega LF Martínez O 2015 How many genes are expressed in a transcriptome? Estimation and results for RNA-Seq. PLoS ONE 10 e0130262 (10.1371/journal.pone.0130262) 26107654 PMC4479379

[bib9] Arzouni AA Vargas-Seymour A Rackham CL Dhadda P Huang GC Choudhary P Nardi N King AJF Jones PM 2017 Mesenchymal stromal cells improve human islet function through released products and extracellular matrix. Clinical Science 131 2835–2845. (10.1042/CS20171251) 29101297

[bib10] Ashcroft FM Harrison DE Ashcroft SJ 1984 Glucose induces closure of single potassium channels in isolated rat pancreatic beta-cells. Nature 312 446–448. (10.1038/312446a0) 6095103

[bib11] Aslanidi OV Mornev OA Skyggebjerg O Arkhammar P Thastrup O Sørensen MP Christiansen PL Conradsen K Scott AC 2001 Excitation wave propagation as a possible mechanism for signal transmission in pancreatic islets of Langerhans. Biophysical Journal 80 1195–1209. (10.1016/S0006-3495(01)76096-1) 11222284 PMC1301315

[bib12] Avrahami D Klochendler A Dor Y Glaser B 2017 Beta cell heterogeneity: an evolving concept. Diabetologia 60 1363–1369. (10.1007/s00125-017-4326-z) 28597073 PMC5554543

[bib13] Bader E Migliorini A Gegg M Moruzzi N Gerdes J Roscioni SS Bakhti M Brandl E Irmler M Beckers J, ***et al*** 2016 Identification of proliferative and mature beta-cells in the islets of Langerhans. Nature 535 430–434. (10.1038/nature18624) 27398620

[bib14] Baggio LL Drucker DJ 2007 Biology of incretins: GLP-1 and GIP. Gastroenterology 132 2131–2157. (10.1053/j.gastro.2007.03.054) 17498508

[bib15] Baron M Veres A Wolock SL Faust AL Gaujoux R Vetere A Ryu JH Wagner BK Shen-Orr SS Klein AM, ***et al*** 2016 A single-cell transcriptomic map of the human and mouse pancreas reveals inter- and intra-cell population structure. Cell Systems 3 346.e344–360.e344. (10.1016/j.cels.2016.08.011) 27667365 PMC5228327

[bib17] Benninger RK Piston DW 2014 Cellular communication and heterogeneity in pancreatic islet insulin secretion dynamics. Trends in Endocrinology and Metabolism 25 399–406. (10.1016/j.tem.2014.02.005) 24679927 PMC4112137

[bib18] Benninger RK Zhang M Head WS Satin LS Piston DW 2008 Gap junction coupling and calcium waves in the pancreatic islet. Biophysical Journal 95 5048–5061. (10.1529/biophysj.108.140863) 18805925 PMC2586567

[bib16] Benninger RK Hutchens T Head WS McCaughey MJ Zhang M Le Marchand SJ Satin LS Piston DW 2014 Intrinsic islet heterogeneity and gap junction coupling determine spatiotemporal Ca(2)(+) wave dynamics. Biophysical Journal 107 2723–2733. (10.1016/j.bpj.2014.10.048) 25468351 PMC4255172

[bib19] Blum B Hrvatin S Schuetz C Bonal C Rezania A Melton DA 2012 Functional beta-cell maturation is marked by an increased glucose threshold and by expression of urocortin 3. Nature Biotechnology 30 261–264. (10.1038/nbt.2141) PMC4617627 22371083

[bib20] Bosco D Armanet M Morel P Niclauss N Sgroi A Muller YD Giovannoni L Parnaud G Berney T 2010 Unique arrangement of alpha- and beta-cells in human islets of Langerhans. Diabetes 59 1202–1210. (10.2337/db09-1177) 20185817 PMC2857900

[bib21] Briant LJB Reinbothe TM Spiliotis I Miranda C Rodriguez B Rorsman P 2017 delta-cells and beta-cells are electrically coupled and regulate alpha-cell activity via somatostatin. Journal of Physiology 596 197–215. (10.1113/JP274581) 28975620 PMC5767697

[bib27] Broichhagen J Schönberger M Cork SC Frank JA Marchetti P Bugliani M Shapiro AMJ Trapp S Rutter GA Hodson DJ, ***et al*** 2014 Optical control of insulin release using a photoswitchable sulfonylurea. Nature Communications 5 5116 (10.1038/ncomms6116) PMC4208094 25311795

[bib22] Broichhagen J Damijonaitis A Levitz J Sokol KR Leippe P Konrad D Isacoff EY Trauner D 2015a Orthogonal optical control of a G protein-coupled receptor with a SNAP-tethered photochromic ligand. ACS Central Science 1 383–393. (10.1021/acscentsci.5b00260) 27162996 PMC4827557

[bib23] Broichhagen J Frank JA Johnston NR Mitchell RK Šmid K Marchetti P Bugliani M Rutter GA Trauner D Hodson DJ 2015b A red-shifted photochromic sulfonylurea for the remote control of pancreatic beta cell function. Chemical Communications 51 6018–6021. (10.1039/C5bib1224D) 25744824 PMC6101206

[bib24] Broichhagen J Frank JA Trauner D 2015c A roadmap to success in photopharmacology. Accounts of Chemical Research 51 6018–6021. 10.1021/acs.accounts.5b00129 26103428

[bib26] Broichhagen J Podewin T Meyer-Berg H von Ohlen Y Johnston NR Jones BJ Bloom SR Rutter GA Hoffmann-Röder A Hodson DJ, ***et al*** 2015d Optical control of insulin secretion using an incretin switch. Angewandte Chemie International Edition 54 15565–15569. (10.1002/anie.201506384) 26585495 PMC4736448

[bib25] Broichhagen J Johnston NR von Ohlen Y Meyer-Berg H Jones BJ Bloom SR Rutter GA Trauner D Hodson DJ 2016 Allosteric optical control of a class B G-protein-coupled receptor. Angewandte Chemie International Edition 55 5865–5868. (10.1002/anie.201600957) 27059784 PMC5031193

[bib28] Bruning D Reckers K Drain P Rustenbeck I 2017 Glucose but not KCl diminishes submembrane granule turnover in mouse beta-cells. Journal of Molecular Endocrinology 59 311–324. (10.1530/JME-17-0063) 28765259

[bib29] Cabrera O Berman DM Kenyon NS Ricordi C Berggren PO Caicedo A 2006 The unique cytoarchitecture of human pancreatic islets has implications for islet cell function. PNAS 103 2334–2339. (10.1073/pnas.0510790103) 16461897 PMC1413730

[bib30] Campbell JE Drucker DJ 2013 Pharmacology, physiology, and mechanisms of incretin hormone action. Cell Metabolism 17 819–837. (10.1016/j.cmet.2013.04.008) 23684623

[bib31] Carrano AC Mulas F Zeng C Sander M 2017 Interrogating islets in health and disease with single-cell technologies. Molecular Metabolism 6 991–1001. (10.1016/j.molmet.2017.04.012) 28951823 PMC5605723

[bib32] Crane A Aguilar-Bryan L 2004 Assembly, maturation, and turnover of KATP channel subunits. Journal of Biological Chemistry 279 9080–9090. (10.1074/jbc.M311079200) 14699091

[bib33] De Vos A Heimberg H Quartier E Huypens P Bouwens L Pipeleers D Schuit F 1995 Human and rat beta cells differ in glucose transporter but not in glucokinase gene expression. Journal of Clinical Investigation 96 2489–2495. (10.1172/JCI118308) 7593639 PMC185903

[bib34] Dean PM Matthews EK 1968 Electrical activity in pancreatic islet cells. Nature 219 389–390. (10.1038/219389a0) 4873864

[bib35] Diakogiannaki E Gribble FM Reimann F 2012 Nutrient detection by incretin hormone secreting cells. Physiology and Behavior 106 387–393. (10.1016/j.physbeh.2011.12.001) 22182802 PMC3361765

[bib36] Diez JA Arrojo EDR Zheng X Stelmashenko OV Chua M Rodriguez-Diaz R Fukuda M Kohler M Leibiger I Tun SBB, ***et al*** 2017 Pancreatic islet blood flow dynamics in primates. Cell Reports 20 1490–1501. (10.1016/j.celrep.2017.07.039) 28793270 PMC5575201

[bib37] Do OH Low JT Gaisano HY Thorn P 2014 The secretory deficit in islets from db/db mice is mainly due to a loss of responding beta cells. Diabetologia 57 1400–1409. (10.1007/s00125-014-3226-8) 24705605 PMC4052007

[bib38] Dorrell C Schug J Canaday PS Russ HA Tarlow BD Grompe MT Horton T Hebrok M Streeter PR Kaestner KH, ***et al*** 2016 Human islets contain four distinct subtypes of β cells. Nature Communications 7 11756 (10.1038/ncomms11756) PMC4942571 27399229

[bib39] Dyachok O Isakov Y Sagetorp J Tengholm A 2006 Oscillations of cyclic AMP in hormone-stimulated insulin-secreting beta-cells. Nature 439 349–352. (10.1038/nature04410) 16421574

[bib40] Ezzati M 2004 Comparative Quantification of Health Risks: Global and Regional Burden of Disease Attributable to Selected Major Risk Factors. WHO: Geneva

[bib41] Farnsworth NL Walter RL Hemmati A Westacott MJ Benninger RK 2015 Low level pro-inflammatory cytokines decrease connexin36 gap junction coupling in mouse and human islets through nitric oxide mediated protein kinase Cdelta. Journal of Biological Chemistry 291 3184–3196. (https;//doi.org/10.1074/jbc.M115.679506) 26668311 10.1074/jbc.M115.679506 PMC4751367

[bib42] Featherstone K Harper CV McNamara A Semprini S Spiller DG McNeilly J McNeilly AS Mullins JJ White MR Davis JR 2011 Pulsatile patterns of pituitary hormone gene expression change during development. Journal of Cell Science 124 3484–3491. (10.1242/jcs.088500) 21984812 PMC3196859

[bib43] Featherstone K Hey K Momiji H McNamara AV Patist AL Woodburn J Spiller DG Christian HC McNeilly AS Mullins JJ, ***et al*** 2016 Spatially coordinated dynamic gene transcription in living pituitary tissue. Elife 5 e08494. 26828110 10.7554/eLife.08494 PMC4749562

[bib44] Forbes JM Cooper ME 2013 Mechanisms of diabetic complications. Physiological Reviews 93 137–188. (10.1152/physrev.00045.2011) 23303908

[bib45] Fowler MJ 2007 Diabetes treatment, part 2: oral agents for glycemic management. Clinical Diabetes 25 131–134. (10.2337/diaclin.25.4.131)

[bib46] Frank JA Yushchenko D Fine NHF Duca M Citir M Broichhagen J Hodson DJ Schultz C Trauner D 2017 Optical control of GPR40 signalling in pancreatic β-cells. Chemical Science 8 7604–7610. (10.1039/C7Sbib1475A) 29568424 PMC5848828

[bib47] Gerdes JM Christou-Savina S Xiong Y Moede T Moruzzi N Karlsson-Edlund P Leibiger B Leibiger IB Ostenson CG Beales PL, ***et al*** 2014 Ciliary dysfunction impairs beta-cell insulin secretion and promotes development of type 2 diabetes in rodents. Nature Communications 5 5308 (10.1038/ncomms6308) 25374274

[bib48] German MS 1993 Glucose sensing in pancreatic islet beta cells: the key role of glucokinase and the glycolytic intermediates. PNAS 90 1781–1785. (10.1073/pnas.90.5.1781) 8446591 PMC45963

[bib49] Giesen C Wang HAO Schapiro D Zivanovic N Jacobs A Hattendorf B Schüffler PJ Grolimund D Buhmann JM Brandt S, ***et al*** 2014 Highly multiplexed imaging of tumor tissues with subcellular resolution by mass cytometry. Nature Methods 11 417–422. (10.1038/nmeth.2869) 24584193

[bib50] Gromada J Brock B Schmitz O Rorsman P 2004 Glucagon-like peptide-1: regulation of insulin secretion and therapeutic potential. Basic and Clinical Pharmacology and Toxicology 95 252–262. (10.1111/j.1742-7843.2004.bib1-1-pto950502.x) 15569269

[bib51] Gutierrez GD Gromada J Sussel L 2017 Heterogeneity of the pancreatic beta cell. Frontiers in Genetics 8 22. 28321233 10.3389/fgene.2017.00022 PMC5337801

[bib52] Halban PA Polonsky KS Bowden DW Hawkins MA Ling C Mather KJ Powers AC Rhodes CJ Sussel L Weir GC 2014 β-Cell failure in type 2 diabetes: postulated mechanisms and prospects for prevention and treatment. Diabetes Care 37 1751–1758. (10.2337/dc14-0396) 24812433 PMC4179518

[bib53] Hang Y Yamamoto T Benninger RKP Brissova M Guo M Bush W Piston DW Powers AC Magnuson M Thurmond DC, ***et al*** 2014 The MafA transcription factor becomes essential to islet -cells soon after birth. Diabetes 63 1994–2005. (10.2337/db13-1001) 24520122 PMC4030115

[bib54] Hanna ST Pigeau GM Galvanovskis J Clark A Rorsman P MacDonald PE 2009 Kiss-and-run exocytosis and fusion pores of secretory vesicles in human beta-cells. Pflugers Archiv 457 1343–1350. (10.1007/s00424-008-0588-0) 18795319

[bib55] Head WS Orseth ML Nunemaker CS Satin LS Piston DW Benninger RK 2012 Connexin-36 gap junctions regulate in vivo first- and second-phase insulin secretion dynamics and glucose tolerance in the conscious mouse. Diabetes 61 1700–1707. (10.2337/db11-1312) 22511206 PMC3379660

[bib56] Henquin JC 2000 Triggering and amplifying pathways of regulation of insulin secretion by glucose. Diabetes 49 1751–1760. (10.2337/diabetes.49.11.1751) 11078440

[bib57] Hiriart M Ramirez-Medeles MC 1991 Functional subpopulations of individual pancreatic B-cells in culture. Endocrinology 128 3193–3198. (10.1210/endo-128-6-3193) 2036985

[bib58] Hoboth P Muller A Ivanova A Mziaut H Dehghany J Sonmez A Lachnit M Meyer-Hermann M Kalaidzidis Y Solimena M 2015 Aged insulin granules display reduced microtubule-dependent mobility and are disposed within actin-positive multigranular bodies. PNAS 112 E667–E676. (10.1073/pnas.1409542112) 25646459 PMC4343180

[bib61] Hodson DJ Schaeffer M Romano N Fontanaud P Lafont C Birkenstock J Molino F Christian H Lockey J Carmignac D, ***et al*** 2012 Existence of long-lasting experience-dependent plasticity in endocrine cell networks. Nature Communications 3 605 (10.1038/ncomms1612) PMC3272579 22215080

[bib59] Hodson DJ Mitchell RK Bellomo EA Sun G Vinet L Meda P Li D Li WH Bugliani M Marchetti P, ***et al*** 2013 Lipotoxicity disrupts incretin-regulated human beta cell connectivity. Journal of Clinical Investigation 123 4182–4194. (10.1172/JCI68459) 24018562 PMC4382273

[bib60] Hodson DJ Mitchell RK Marselli L Pullen TJ Gimeno Brias S Semplici F Everett KL Cooper DM Bugliani M Marchetti P, ***et al*** 2014 ADCY5 couples glucose to insulin secretion in human islets. Diabetes 63 3009–3021. (10.2337/db13-1607) 24740569 PMC4141364

[bib62] Home P Riddle M Cefalu WT Bailey CJ Bretzel RG del Prato S Leroith D Schernthaner G van Gaal L Raz I 2014 Insulin therapy in people with type 2 diabetes: opportunities and challenges? Diabetes Care 37 1499–1508. (10.2337/dc13-2743) 24855154 PMC5131884

[bib63] Ibanez AJ Fagerer SR Schmidt AM Urban PL Jefimovs K Geiger P Dechant R Heinemann M Zenobi R 2013 Mass spectrometry-based metabolomics of single yeast cells. PNAS 110 8790–8794. (10.1073/pnas.1209302110) 23671112 PMC3670324

[bib64] Jetton TL Magnuson MA 1992 Heterogeneous expression of glucokinase among pancreatic beta cells. PNAS 89 2619–2623. (10.1073/pnas.89.7.2619) 1557365 PMC48713

[bib66] Johnson JD Ahmed NT Luciani DS Han Z Tran H Fujita J Misler S Edlund H Polonsky KS 2003 Increased islet apoptosis in Pdx1+/- mice. Journal of Clinical Investigation 111 1147–1160. (10.1172/JCI200316537) 12697734 PMC152933

[bib67] Johnson JS Kono T Tong X Yamamoto WR Zarain-Herzberg A Merrins MJ Satin LS Gilon P Evans-Molina C 2014 Pancreatic and duodenal homeobox protein 1 (Pdx-1) maintains endoplasmic reticulum calcium levels through transcriptional regulation of sarco-endoplasmic reticulum calcium ATPase 2b (SERCA2b) in the islet beta cell. Journal of Biological Chemistry 289 32798–32810. (10.1074/jbc.M114.575191) 25271154 PMC4239629

[bib65] Johnson CH Ivanisevic J Siuzdak G 2016 Metabolomics: beyond biomarkers and towards mechanisms. Nature Reviews Molecular Cell Biology 17 451–459. (10.1038/nrm.2016.25) PMC5729912 26979502

[bib68] Johnston Natalie R Mitchell Ryan K Haythorne E Pessoa Maria P Semplici F Ferrer J Piemonti L Marchetti P Bugliani M Bosco D, ***et al*** 2016 Beta cell hubs dictate pancreatic islet responses to glucose. Cell Metabolism 24 389–401. (10.1016/j.cmet.2016.06.020) 27452146 PMC5031557

[bib69] Kenty JH Melton DA 2015 Testing pancreatic islet function at the single cell level by calcium influx with associated marker expression. PLoS ONE 10 e0122044 (10.1371/journal.pone.0122044) 25853429 PMC4390334

[bib70] Kiekens R In’t Veld P Mahler T Schuit F Van De Winkel M Pipeleers D 1992 Differences in glucose recognition by individual rat pancreatic B cells are associated with intercellular differences in glucose-induced biosynthetic activity. Journal of Clinical Investigation 89 117–125. (10.1172/JCI115551) 1729264 PMC442826

[bib71] Klochendler A Caspi I Corem N Moran M Friedlich O Elgavish S Nevo Y Helman A Glaser B Eden A, ***et al*** 2016 The genetic program of pancreatic beta-cell replication in vivo. Diabetes 65 2081–2093. (10.2337/db16-0003) 26993067 PMC4915587

[bib72] Konstantinova I Nikolova G Ohara-Imaizumi M Meda P Kucera T Zarbalis K Wurst W Nagamatsu S Lammert E 2007 EphA-Ephrin-A-mediated beta cell communication regulates insulin secretion from pancreatic islets. Cell 129 359–370. (10.1016/j.cell.2007.02.044) 17448994

[bib73] Kullmann S Heni M Hallschmid M Fritsche A Preissl H Haring HU 2016 Brain insulin resistance at the crossroads of metabolic and cognitive disorders in humans. Physiological Reviews 96 1169–1209. (10.1152/physrev.00032.2015) 27489306

[bib74] Kushibiki T Okawa S Hirasawa T Ishihara M 2015 Optogenetic control of insulin secretion by pancreatic β-cells in vitro and in vivo. Gene Therapy 22 553–559. (10.1038/gt.2015.23) 25809465

[bib75] Kwan EP Gaisano HY 2009 Rescuing the subprime meltdown in insulin exocytosis in diabetes. Annals of the New York Academy of Sciences 1152 154–164. (10.1111/j.1749-6632.2008.03990.x) 19161386

[bib76] Lawlor N George J Bolisetty M Kursawe R Sun L Sivakamasundari V Kycia I Robson P Stitzel ML 2017 Single-cell transcriptomes identify human islet cell signatures and reveal cell-type–specific expression changes in type 2 diabetes. Genome Research 27 208–222. (10.1101/gr.212720.116) 27864352 PMC5287227

[bib77] Le Tissier P Campos P Lafont C Romanò N Hodson DJ Mollard P 2016 An updated view of hypothalamic–vascular–pituitary unit function and plasticity. Nature Reviews Endocrinology 13 257–267. 10.1038/nrendo.2016.193 27934864

[bib78] Leech CA Dzhura I Chepurny OG Kang G Schwede F Genieser HG Holz GG 2011 Molecular physiology of glucagon-like peptide-1 insulin secretagogue action in pancreatic beta cells. Progress in Biophysics and Molecular Biology 107 236–247. (10.1016/j.pbiomolbio.2011.07.005) 21782840 PMC3200499

[bib79] Leippe P Koehler Leman J Trauner D 2017 Specificity and speed: tethered photopharmacology. Biochemistry 56 5214–5220. (10.1021/acs.biochem.7b00687) 28876905

[bib80] Lernmark A 1974 The preparation of, and studies on, free cell suspensions from mouse pancreatic islets. Diabetologia 10 431–438. (10.1007/BF01221634) 4375640

[bib81] Levitz J Broichhagen J Leippe P Konrad D Trauner D Isacoff EY 2017 Dual optical control and mechanistic insights into photoswitchable group II and III metabotropic glutamate receptors. PNAS 114 3546–3554. (10.1073/pnas.1619652114) PMC5410775 28396447

[bib82] Li J Shuai HY Gylfe E Tengholm A 2013 Oscillations of sub-membrane ATP in glucose-stimulated beta cells depend on negative feedback from Ca(2+). Diabetologia 56 1577–1586. (10.1007/s00125-013-2894-0) 23536115 PMC3671113

[bib83] Lovatt D Ruble BK Lee J Dueck H Kim TK Fisher S Francis C Spaethling JM Wolf JA Grady MS, ***et al*** 2014 Transcriptome in vivo analysis (TIVA) of spatially defined single cells in live tissue. Nature Methods 11 190–196. (10.1038/nmeth.2804) 24412976 PMC3964595

[bib84] Low JT Mitchell JM Do OH Bax J Rawlings A Zavortink M Morgan G Parton RG Gaisano HY Thorn P 2013 Glucose principally regulates insulin secretion in mouse islets by controlling the numbers of granule fusion events per cell. Diabetologia 56 2629–2637. (10.1007/s00125-013-3019-5) 23995471 PMC3825531

[bib85] Low JT Zavortink M Mitchell JM Gan WJ Do OH Schwiening CJ Gaisano HY Thorn P 2014 Insulin secretion from beta cells in intact mouse islets is targeted towards the vasculature. Diabetologia 57 1655–1663. (10.1007/s00125-014-3252-6) 24795086 PMC4079948

[bib86] Lynn FC Pamir N Ng EHC McIntosh CHS Kieffer TJ Pederson RA 2001 Defective glucose-dependent insulinotropic polypeptide receptor expression in diabetic fatty Zucker rats. Diabetes 50 1004–1011. (10.2337/diabetes.50.5.1004) 11334402

[bib87] Ma L Bindokas VP Kuznetsov A Rhodes C Hays L Edwardson JM Ueda K Steiner DF Philipson LH 2004 Direct imaging shows that insulin granule exocytosis occurs by complete vesicle fusion. PNAS 101 9266–9271. (10.1073/pnas.0403201101) 15197259 PMC438965

[bib89] MacDonald PE El-Kholy W Riedel MJ Salapatek AM Light PE Wheeler MB 2002 The multiple actions of GLP-1 on the process of glucose-stimulated insulin secretion. Diabetes 51 (Supplement 3) S434–S442. (10.2337/diabetes.51.2007.S434) 12475787

[bib88] MacDonald PE Braun M Galvanovskis J Rorsman P 2006 Release of small transmitters through kiss-and-run fusion pores in rat pancreatic beta cells. Cell Metabolism 4 283–290. (10.1016/j.cmet.2006.08.011) 17011501

[bib90] Maedler K Aguayo-Mazzucato C Sanchez-Soto C Godinez-Puig V Gutiérrez-Ospina G Hiriart M 2006 Restructuring of pancreatic islets and insulin secretion in a postnatal critical window. PLoS ONE 1 e35 (10.1371/journal.pone.0000035) 17183663 PMC1762382

[bib91] Martin F Soria B 1996 Glucose-induced [Ca2+]i oscillations in single human pancreatic islets. Cell Calcium 20 409–414. (10.1016/S0143-4160(96)90003-2) 8955555

[bib92] Mehta ZB Johnston NR Nguyen-Tu MS Broichhagen J Schultz P Larner DP Leclerc I Trauner D Rutter GA Hodson DJ 2017 Remote control of glucose homeostasis in vivo using photopharmacology. Scientific Reports 7 291 (10.1038/s41598-017-00397-0) 28331198 PMC5428208

[bib93] Meier JJ Nauck MA 2010 Is the diminished incretin effect in type 2 diabetes just an epi-phenomenon of impaired beta-cell function? Diabetes 59 1117–1125. (10.2337/db09-1899) 20427697 PMC2857890

[bib94] Michau A Hodson DJ Fontanaud P Guillou A Espinosa-Carrasco G Molino F Peters CJ Robinson IC Le Tissier P Mollard P, ***et al*** 2015 Metabolism regulates exposure of pancreatic islets to circulating molecules in vivo. Diabetes 65 463–475. (10.2337/db15-1168) 26581596

[bib95] Mitchell RK Mondragon A Chen L McGinty JA French PM Ferrer J Thorens B Hodson DJ Rutter GA Xavier GD 2014 Selective disruption of Tcf7l2 in the pancreatic beta cell impairs secretory function and lowers beta cell mass. Human Molecular Genetics 24 1390–1399. (10.1093/hmg/ddu553) 25355422 PMC4321446

[bib96] Mojibian M Harder B Hurlburt A Bruin JE Asadi A Kieffer TJ 2013 Implanted islets in the anterior chamber of the eye are prone to autoimmune attack in a mouse model of diabetes. Diabetologia 56 2213–2221. (10.1007/s00125-013-3004-z) 23933952

[bib97] Muraro MJ Dharmadhikari G Grun D Groen N Dielen T Jansen E van Gurp L Engelse MA Carlotti F de Koning EJ, ***et al*** 2016 A single-cell transcriptome atlas of the human pancreas. Cell Systems 3 385.e383–394.e383. (10.1016/j.cels.2016.09.002) 27693023 PMC5092539

[bib98] Nauck MA Homberger E Siegel EG Allen RC Eaton RP Ebert R Creutzfeldt W 1986 Incretin effects of increasing glucose loads in man calculated from venous insulin and C-peptide responses. Journal of Clinical Endocrinology and Metabolism 63 492–498. (10.1210/jcem-63-2-492) 3522621

[bib99] Nauck MA Meier JJ 2016 The incretin effect in healthy individuals and those with type 2 diabetes: physiology, pathophysiology, and response to therapeutic interventions. Lancet Diabetes and Endocrinology 4 525–536. (10.1016/S2213-8587(15)00482-9) 26876794

[bib101] Nyman LR Wells KS Head WS McCaughey M Ford E Brissova M Piston DW Powers AC 2008 Real-time, multidimensional in vivo imaging used to investigate blood flow in mouse pancreatic islets. Journal of Clinical Investigation 118 3790–3797. (10.1172/JCI36209) 18846254 PMC2564611

[bib100] Nyman LR Ford E Powers AC Piston DW 2010 Glucose-dependent blood flow dynamics in murine pancreatic islets in vivo. American Journal of Physiology: Endocrinology and Metabolism 298 E807–E814. (10.1152/ajpendo.00715.2009) 20071562 PMC2853211

[bib150] Ozsolak F Milos PM 2011 RNA sequencing: advances, challenges and opportunities. Nature Reviews Genetics 12 87–98. (10.1038/nrg2934) PMC3031867 21191423

[bib102] Parker HE Wallis K le Roux CW Wong KY Reimann F Gribble FM 2012 Molecular mechanisms underlying bile acid-stimulated glucagon-like peptide-1 secretion. British Journal of Pharmacology 165 414–423. (10.1111/j.1476-5381.2011.01561.x) 21718300 PMC3268195

[bib103] Paschen M Moede T Leibiger B Jacob S Bryzgalova G Leibiger IB Berggren PO 2016 Non-invasive cell type selective in vivo monitoring of insulin resistance dynamics. Scientific Reports 6 21448 (10.1038/srep21448) 26899548 PMC4761884

[bib104] Piston DW Knobel SM Postic C Shelton KD Magnuson MA 1999 Adenovirus-mediated knockout of a conditional glucokinase gene in isolated pancreatic islets reveals an essential role for proximal metabolic coupling events in glucose-stimulated insulin secretion. Journal of Biological Chemistry 274 1000–1004. (10.1074/jbc.274.2.1000) 9873043

[bib105] Podewin T Ast J Broichhagen J Fine NHF Nasteska D Leippe P Gailer M Buenaventura T Kanda N Jones BJ, ***et al*** 2018 Conditional and reversible activation of class A and B G protein-coupled receptors using tethered pharmacology. ACS Central Science 4 166–179. (10.1021/acscentsci.7b00237) 29532016 PMC5832994

[bib106] Porte D Kahn SE 2001 Beta-cell dysfunction and failure in type 2 diabetes: potential mechanisms. Diabetes 50 S160–S163. (10.2337/diabetes.50.2007.S160) 11272181

[bib107] Prentki M Nolan CJ 2006 Islet beta cell failure in type 2 diabetes. Journal of Clinical Investigation 116 1802–1812. (10.1172/JCI29103) 16823478 PMC1483155

[bib151] Proserpio V Lönnberg T 2016 Single‐cell technologies are revolutionizing the approach to rare cells. Immunology & Cell Biology 94 225–229. (10.1038/icb.2015.106) 26620630 PMC4796591

[bib108] Raimondo JV Kay L Ellender TJ Akerman CJ 2012 Optogenetic silencing strategies differ in their effects on inhibitory synaptic transmission. Nature Neuroscience 15 1102–1104. (10.1038/nn.3143) 22729174 PMC3428858

[bib110] Ravier MA Sehlin J Henquin JC 2002 Disorganization of cytoplasmic Ca(2+) oscillations and pulsatile insulin secretion in islets from ob/ obmice. Diabetologia 45 1154–1163. (10.1007/s00125-002-0883-9) 12189446

[bib109] Ravier MA Guldenagel M Charollais A Gjinovci A Caille D Sohl G Wollheim CB Willecke K Henquin JC Meda P 2005 Loss of connexin36 channels alters beta-cell coupling, islet synchronization of glucose-induced Ca2+ and insulin oscillations, and basal insulin release. Diabetes 54 1798–1807. (10.2337/diabetes.54.6.1798) 15919802

[bib111] Reinbothe TM Safi F Axelsson AS Mollet IG Rosengren AH 2014 Optogenetic control of insulin secretion in intact pancreatic islets with beta-cell-specific expression of Channelrhodopsin-2. Islets 6 e28095 (10.4161/isl.28095) 25483880 PMC4593566

[bib112] Rodriguez-Diaz R Speier S Molano RD Formoso A Gans I Abdulreda MH Cabrera O Molina J Fachado A Ricordi C, ***et al*** 2012 Noninvasive in vivo model demonstrating the effects of autonomic innervation on pancreatic islet function. PNAS 109 21456–21461. (10.1073/pnas.1211659110) 23236142 PMC3535593

[bib113] Rorsman P Braun M 2013 Regulation of insulin secretion in human pancreatic islets. Annual Review of Physiology 75 155–179. (10.1146/annurev-physiol-030212-183754) 22974438

[bib114] Roscioni SS Migliorini A Gegg M Lickert H 2016 Impact of islet architecture on beta-cell heterogeneity, plasticity and function. Nature Reviews: Endocrinology 12 695–709. (10.1038/nrendo.2016.147) 27585958

[bib115] Rutter GA Hill EV 2006 Insulin vesicle release: walk, kiss, pause … then run. Physiology 21 189–196. (10.1152/physiol.00002.2006) 16714477

[bib116] Rutter GA Hodson DJ 2013 Minireview: intraislet regulation of insulin secretion in humans. Molecular Endocrinology 27 1984–1995. (10.1210/me.2013-1278) 24243488 PMC5426601

[bib117] Rutter GA Hodson DJ 2014 Beta cell connectivity in pancreatic islets: a type 2 diabetes target? Cellular and Molecular Life Sciences 72 453–467. (10.1007/s00018-014-1755-4) 25323131 PMC11113448

[bib118] Rutter GA Pullen TJ Hodson DJ Martinez-Sanchez A 2015 Pancreatic beta-cell identity, glucose sensing and the control of insulin secretion. Biochemical Journal 466 203–218. (10.1042/BJ20141384) 25697093

[bib119] Sachdeva MM Claiborn KC Khoo C Yang J Groff DN Mirmira RG Stoffers DA 2009 Pdx1 (MODY4) regulates pancreatic beta cell susceptibility to ER stress. PNAS 106 19090–19095. (10.1073/pnas.0904849106) 19855005 PMC2776433

[bib120] Salomon D Meda P 1986 Heterogeneity and contact-dependent regulation of hormone secretion by individual B cells. Experimental Cell Research 162 507–520. (10.1016/0014-4827(86)90354-X) 3510882

[bib121] Samuel Varman T Shulman Gerald I 2012 Mechanisms for insulin resistance: common threads and missing links. Cell 148 852–871. (10.1016/j.cell.2012.02.017) 22385956 PMC3294420

[bib122] Sanchez-Cardenas C Fontanaud P He Z Lafont C Meunier AC Schaeffer M Carmignac D Molino F Coutry N Bonnefont X, ***et al*** 2010 Pituitary growth hormone network responses are sexually dimorphic and regulated by gonadal steroids in adulthood. PNAS 107 21878–21883. (10.1073/pnas.1010849107) 21098290 PMC3003007

[bib123] Schuit FC In’t Veld PA Pipeleers DG 1988 Glucose stimulates proinsulin biosynthesis by a dose-dependent recruitment of pancreatic beta cells. PNAS 85 3865–3869. (10.1073/pnas.85.11.3865) 3287379 PMC280320

[bib124] Serre-Beinier V Le Gurun S Belluardo N Trovato-Salinaro A Charollais A Haefliger JA Condorelli DF Meda P 2000 Cx36 preferentially connects beta-cells within pancreatic islets. Diabetes 49 727–734. (10.2337/diabetes.49.5.727) 10905480

[bib125] Shields BC Kahuno E Kim C Apostolides PF Brown J Lindo S Mensh BD Dudman JT Lavis LD Tadross MR 2017 Deconstructing behavioral neuropharmacology with cellular specificity. Science 356 eaaj2161 (10.1126/science.aaj2161) 28385956

[bib126] Singh SP Janjuha S Hartmann T Kayisoglu O Konantz J Birke S Murawala P Alfar EA Murata K Eugster A, ***et al*** 2017 Different developmental histories of beta-cells generate functional and proliferative heterogeneity during islet growth. Nature Communications 8 664 (10.1038/s41467-017-00461-3) PMC5610262 28939870

[bib127] Speier S Nyqvist D Cabrera O Yu J Molano RD Pileggi A Moede T Kohler M Wilbertz J Leibiger B, ***et al*** 2008a Noninvasive in vivo imaging of pancreatic islet cell biology. Nature Medicine 14 574–578. (10.1038/nm1701) PMC3538807 18327249

[bib128] Speier S Nyqvist D Köhler M Caicedo A Leibiger IB Berggren P-O 2008b Noninvasive high-resolution in vivo imaging of cell biology in the anterior chamber of the mouse eye. Nature Protocols 3 1278–1286. (10.1038/nprot.2008.118) 18714296 PMC3538838

[bib129] Squires PE Persaud SJ Hauge-Evans AC Gray E Ratcliff H Jones PM 2002 Co-ordinated Ca(2+)-signalling within pancreatic islets: does beta-cell entrainment require a secreted messenger. Cell Calcium 31 209–219. (10.1016/S0143-4160(02)00034-9) 12098223

[bib130] Stancill JS Cartailler J-P Clayton HW O’Connor JT Dickerson MT Dadi PK Osipovich AB Jacobson DA Magnuson MA 2017 Chronic β-Cell depolarization impairs β-Cell identity by disrupting a network of Ca 2+ -regulated genes. Diabetes 66 2175–2187. (10.2337/db16-1355) 28550109 PMC5521870

[bib131] Steiner DJ Kim A Miller K Hara M 2010 Pancreatic islet plasticity: interspecies comparison of islet architecture and composition. Islets 2 135–145. (10.4161/isl.2.3.11815) 20657742 PMC2908252

[bib132] Stozer A Dolensek J Rupnik MS 2013 Glucose-stimulated calcium dynamics in islets of Langerhans in acute mouse pancreas tissue slices. PLoS ONE 8 e54638 (10.1371/journal.pone.0054638) 23358454 PMC3554663

[bib133] Szabat M Luciani DS Piret JM Johnson JD 2009 Maturation of adult beta-cells revealed using a Pdx1/insulin dual-reporter lentivirus. Endocrinology 150 1627–1635. (10.1210/en.2008-1224) 19095744

[bib134] Takahashi N Kishimoto T Nemoto T Kadowaki T Kasai H 2002 Fusion pore dynamics and insulin granule exocytosis in the pancreatic islet. Science 297 1349–1352. (10.1126/science.1073806) 12193788

[bib135] Tsuboi T Rutter GA 2003 Multiple forms of “kiss-and-run” exocytosis revealed by evanescent wave microscopy. Current Biology: CB 13 563–567. (10.1016/S0960-9822(03)00176-3) 12676086

[bib136] van der Meulen T Mawla AM DiGruccio MR Adams MW Nies V Dolleman S Liu S Ackermann AM Caceres E Hunter AE, ***et al*** 2017 Virgin beta cells persist throughout life at a neogenic niche within pancreatic islets. Cell Metabolism 25 911.e916–926.e916. (10.1016/j.cmet.2017.03.017) 28380380 PMC8586897

[bib137] Velema WA Szymanski W Feringa BL 2014 Photopharmacology: beyond proof of principle. Journal of the American Chemical Society 136 2178–2191. (10.1021/ja413063e) 24456115

[bib138] Wang YJ Golson Maria L Schug J Traum D Liu C Vivek K Dorrell C Naji A Powers Alvin C Chang K-M, ***et al*** 2016a Single-cell mass cytometry analysis of the human endocrine pancreas. Cell Metabolism 24 616–626. (10.1016/j.cmet.2016.09.007) 27732837 PMC5123805

[bib139] Wang YJ Schug J Won KJ Liu C Naji A Avrahami D Golson ML Kaestner KH 2016b Single-cell transcriptomics of the human endocrine pancreas. Diabetes 65 3028–3038. (10.2337/db16-0405) 27364731 PMC5033269

[bib140] Westacott MJ Farnsworth NL St Clair JR Poffenberger G Heintz A Ludin NW Hart NJ Powers AC Benninger RKP 2017a Age-dependent decline in the coordinated [Ca(2+)] and insulin secretory dynamics in human pancreatic islets. Diabetes 66 2436–2445. (10.2337/db17-0137) 28588099 PMC5566297

[bib141] Westacott MJ Ludin NWF Benninger RKP 2017b Spatially organized beta-cell subpopulations control electrical dynamics across islets of Langerhans. Biophysical Journal 113 1093–1108. (10.1016/j.bpj.2017.07.021) 28877492 PMC5658715

[bib142] Wheeler MB Sheu L Ghai M Bouquillon A Grondin G Weller U Beaudoin AR Bennett MK Trimble WS Gaisano HY 1996 Characterization of SNARE protein expression in beta cell lines and pancreatic islets. Endocrinology 137 1340–1348. (10.1210/endo.137.4.8625909) 8625909

[bib143] Wojtusciszyn A Armanet M Morel P Berney T Bosco D 2008 Insulin secretion from human beta cells is heterogeneous and dependent on cell-to-cell contacts. Diabetologia 51 1843–1852. (10.1007/s00125-008-1103-z) 18665347

[bib144] Xin Y Kim J Ni M Wei Y Okamoto H Lee J Adler C Cavino K Murphy AJ Yancopoulos GD, ***et al*** 2016 Use of the Fluidigm C1 platform for RNA sequencing of single mouse pancreatic islet cells. PNAS 113 3293–3298. (10.1073/pnas.1602306113) 26951663 PMC4812709

[bib145] Yang YH Szabat M Bragagnini C Kott K Helgason CD Hoffman BG Johnson JD 2011 Paracrine signalling loops in adult human and mouse pancreatic islets: netrins modulate beta cell apoptosis signalling via dependence receptors. Diabetologia 54 828–842. (10.1007/s00125-010-2012-5) 21212933

[bib146] Zeng C Mulas F Sui Y Guan T Miller N Tan Y Liu F Jin W Carrano AC Huising MO, ***et al*** 2017 Pseudotemporal ordering of single cells reveals metabolic control of postnatal β cell proliferation. Cell Metabolism 25 1160.e1111–1175.e1111. (10.1016/j.cmet.2017.04.014) 28467932 PMC5501713

[bib147] Zhang M Goforth P Bertram R Sherman A Satin L 2003 The Ca2+ dynamics of isolated mouse beta-cells and islets: implications for mathematical models. Biophysical Journal 84 2852–2870. (10.1016/S0006-3495(03)70014-9) 12719219 PMC1302850

[bib148] Zhang Q Galvanovskis J Abdulkader F Partridge CJ Gopel SO Eliasson L Rorsman P 2008 Cell coupling in mouse pancreatic beta-cells measured in intact islets of Langerhans. Philosophical Transactions: Series A, Mathematical, Physical, and Engineering Sciences 366 3503–3523. (10.1098/rsta.2008.0110) 18632454

[bib149] Zhu S Larkin D Lu S Inouye C Haataja L Anjum A Kennedy R Castle D Arvan P 2016 Monitoring C-peptide storage and secretion in islet β-cells in vitro and in vivo. Diabetes 65 699–709. (10.2337/db15-1264) 26647386 PMC4764152

