# Supplementary material for: The role of beta cell heterogeneity in islet function and insulin release
Source: J Mol Endocrinol. 2018 Apr 16;61(1):R43–60. doi: 10.1530/JME-18-0011 (PMC5976077; doi:10.1530/JME-18-0011)
Supplement: Supporting methods [file jme-60-R43-s001.pdf]

Conventional methodology for understanding beta cell heterogeneity at transcriptomic and protein levels has typically involved next-generation sequencing and mass cytometry (CyTOF). While RNA sequencing (RNA-seq) measures the expression profile averaged across thousands of cells, single cell variants (scRNA-seq) are able to identify subpopulations based on cluster or principal component analyses. Thus, scRNA-seq has provided a powerful tool for understanding beta cell heterogeneity in the rodent and human islet. Along similar lines, fluorescence-activated cell sorting (FACS) approaches allow identification of cell subtypes using specific antibodies. However, the number of available channels for FACS, and hence protein markers that can be screened, is limited by spectral overlap between secondary fluorescent antibodies. Mass cytometry (CyTOF) circumvents this problem by instead relying on time of flight (TOF) of metal-labelled antibodies. Since there is minimal overlap between TOFs, upwards of 40 protein markers can be simultaneously measured. Reporter genes/proteins, generally identified using FACS or CyTOF, can be used to further separate beta cell subpopulations depending on possession of a specific marker or not.

Supplementary Methods: Single-cell screening methodology for understanding beta cell heterogeneity.
